# Traumatic Pseudo-Internuclear Ophthalmoplegia Due to Suspected Medial Rectus Injury Following Orbital Trauma: A Case Report

**DOI:** 10.7759/cureus.107359

**Published:** 2026-04-19

**Authors:** Worapot Srimanan, Sunita Sawangsribanterng, Setthapoom Poolsawat, Phawasutthi Keokajee

**Affiliations:** 1 Ophthalmology, Phramongkutklao Hospital, Bangkok, THA

**Keywords:** internuclear ophthalmoplegia, medial rectus injury, neuro-ophthalmology, orbital trauma, pseudo-ino

## Abstract

Internuclear ophthalmoplegia (INO) is a classic neuro-ophthalmologic syndrome caused by disruption of the medial longitudinal fasciculus (MLF), resulting in impaired adduction of the affected eye and abducting nystagmus of the contralateral eye during horizontal gaze. However, an INO-like ocular motility pattern may occur without an MLF lesion, a phenomenon termed pseudo-internuclear ophthalmoplegia. Peripheral etiologies, including extraocular muscle injury, may produce similar clinical findings and pose a diagnostic challenge. A 34-year-old man presented after a motor vehicle accident with severe head trauma and loss of consciousness. Computed tomography revealed multiple skull fractures and epidural hemorrhage without evidence of brainstem injury. Ophthalmic examination showed limited adduction of the right eye that failed to cross the midline, with abducting nystagmus of the left eye on left gaze, mimicking internuclear ophthalmoplegia. Orbital imaging demonstrated a subtle medial orbital wall fracture without clear medial rectus entrapment. Given the absence of neuroimaging evidence of brainstem pathology and the presence of orbital trauma, traumatic injury to the medial rectus muscle was suspected. The patient was managed conservatively and showed gradual improvement in ocular motility and resolution of diplopia over two weeks. Traumatic pseudo-internuclear ophthalmoplegia is an uncommon but important differential diagnosis in patients presenting with INO-like ocular motility abnormalities after orbital trauma. Careful clinical examination and orbital imaging are essential to distinguish peripheral extraocular muscle injury from central MLF lesions.

## Introduction

Internuclear ophthalmoplegia (INO) is a distinctive ocular motor disorder caused by disruption of the medial longitudinal fasciculus (MLF), a critical neural pathway that coordinates conjugate horizontal eye movements between the abducens nucleus and the contralateral oculomotor nucleus. Clinically, INO is characterized by impaired adduction of the affected eye, accompanied by abducting nystagmus of the contralateral eye during horizontal gaze attempts [1,2]. Convergence is typically preserved because the medial rectus subnucleus remains intact [2].

INO is traditionally regarded as a highly localizing sign of brainstem pathology. The most common etiologies include ischemic stroke in older individuals and demyelinating disorders such as multiple sclerosis in younger patients [1,3]. Less common causes include infection, tumor, inflammatory disorders, and trauma [3]. Because of its strong neuroanatomical localization, the detection of INO generally prompts urgent neuroimaging to evaluate for brainstem lesions. In the setting of trauma, however, distinguishing between central (brainstem) and peripheral (orbital) causes is particularly important, as mislocalization may lead to unnecessary investigations or inappropriate clinical management.

However, not all INO-like ocular motility patterns arise from lesions of the medial longitudinal fasciculus. The term pseudo-internuclear ophthalmoplegia describes clinical presentations that mimic INO but occur without injury to the MLF. These cases typically result from peripheral disorders affecting the extraocular muscles or neuromuscular transmission [4]. Ocular myasthenia gravis is the most commonly reported cause and can produce adduction weakness that closely resembles INO [5,6]. Pseudo-INO may also occur in other systemic neurologic conditions, such as Guillain-Barré syndrome, and has been reported after iatrogenic medial rectus paresis following strabismus surgery [4,7]. In these situations, medial rectus dysfunction leads to increased neural drive to maintain conjugate gaze, which can generate abducting nystagmus in the contralateral eye and simulate the classic INO pattern [4].

Orbital trauma represents another potential but underrecognized cause of pseudo-INO. Trauma may impair extraocular muscle function through mechanisms including contusion, ischemia, entrapment within orbital fractures, or direct muscle rupture [8]. Although orbital floor fractures more commonly involve the inferior rectus muscle, medial orbital wall fractures may affect the medial rectus muscle and produce horizontal diplopia or adduction deficits [9]. Even subtle fractures without clear radiologic entrapment may disrupt medial rectus function due to edema or muscle contusion.

Herein, we describe a patient who developed an INO-like ocular motility pattern following blunt head trauma. Neuroimaging showed no evidence of brainstem pathology but revealed a subtle medial orbital wall fracture. The clinical course and spontaneous improvement suggested traumatic medial rectus dysfunction resulting in pseudo-internuclear ophthalmoplegia. Although pseudo-INO has been described in neuromuscular and iatrogenic conditions, trauma-related cases, particularly those without radiologic evidence of muscle entrapment, remain poorly characterized. This case highlights an important diagnostic pitfall in neuro-ophthalmology: INO-like ocular motility patterns do not always originate from central brainstem lesions and may occasionally arise from peripheral orbital pathology.

## Case presentation

Patient information

A 34-year-old man with a history of methamphetamine-induced psychosis was involved in a high-speed motorcycle accident and presented with loss of consciousness. He was transferred to our tertiary care hospital with an initial Glasgow Coma Scale (GCS) score of E1V1M1 [10]. Due to poor airway protection and severe neurological impairment, emergent endotracheal intubation was performed upon arrival. Following airway stabilization and initial resuscitative measures, his neurological status improved slightly to E1VTM4. Supportive medical management was continued in the intensive care unit, and the patient remained hemodynamically stable.

Initial diagnostic assessment

**Figure 1:**
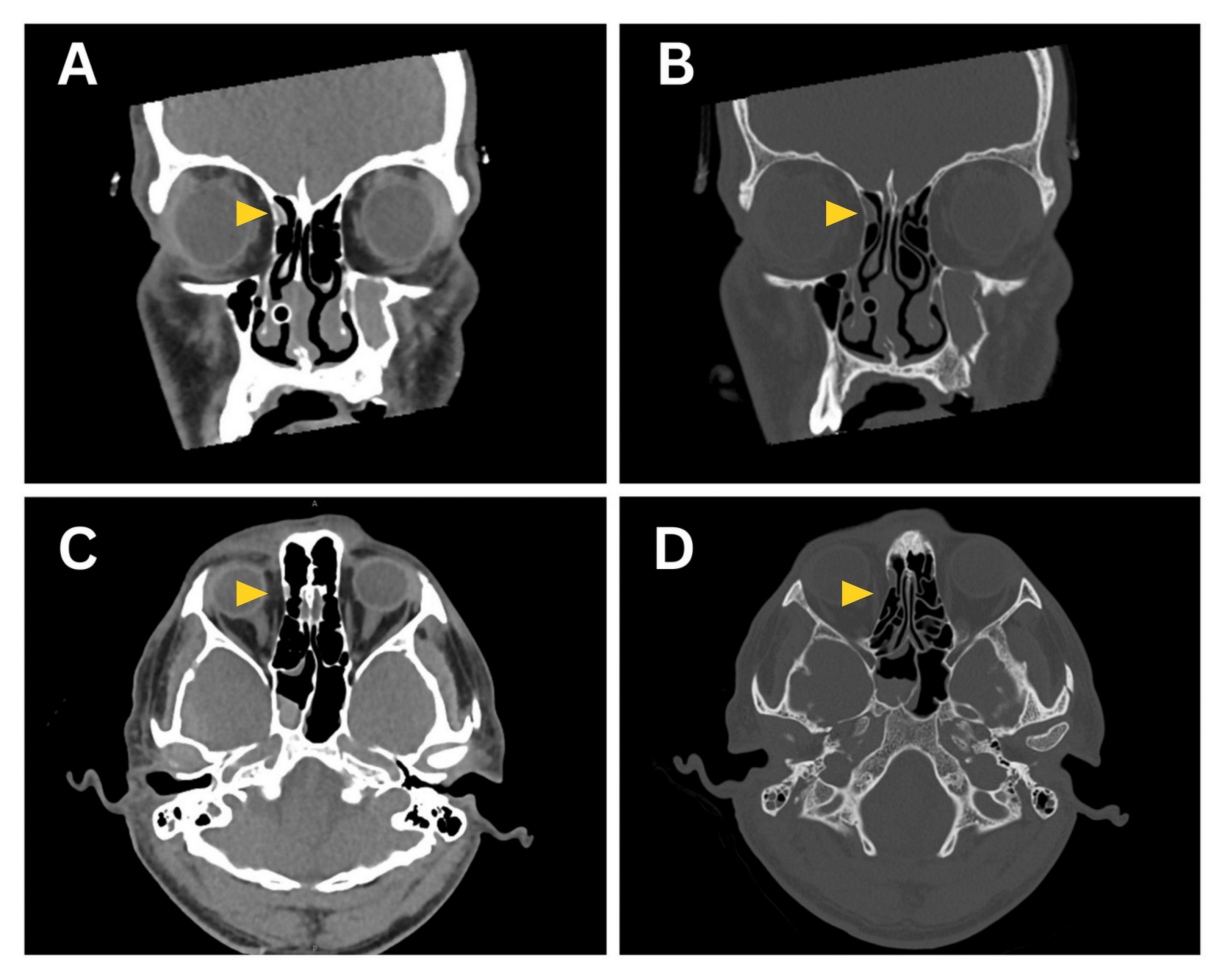
Initial CT imaging of the brain and orbit at presentation. (A-B) Coronal CT sections demonstrate a subtle fracture of the right medial orbital wall (yellow arrowheads) without evidence of medial rectus muscle entrapment. (C-D) Axial CT sections confirm the small medial orbital wall fracture (yellow arrowheads) adjacent to the medial rectus muscle, with preserved extraocular muscle alignment and no radiologic signs of entrapment.

Given the mechanism of injury and altered mental status, non-contrast computed tomography (CT) of the brain and facial bones was performed to evaluate for intracranial hemorrhage and facial fractures. Brain imaging revealed multiple skull fractures and an epidural hematoma involving the right parieto-temporal region and cerebellum. Imaging of the facial bones demonstrated a displaced left zygomaticomaxillary complex (ZMC) fracture, fractures of the nasal bone, a subtle fracture of the medial wall of the right orbit, and fractures involving the orbital floor and lateral wall of the left orbit. Importantly, no extraocular muscle entrapment was detected in association with these fractures. The radiologic findings are summarized in Figure 1.

Because of the complex craniofacial injuries, both the plastic surgery and ophthalmology teams were consulted in the emergency department.

Initial ophthalmic examination

The initial ophthalmologic evaluation was limited by the patient’s decreased level of consciousness, which prevented a formal assessment of best-corrected visual acuity (BCVA). Examination revealed periorbital swelling and ecchymosis with hemorrhagic chemosis in the left eye. Hertel exophthalmometry with a base of 106 mm measured 13 mm in the right eye and 14 mm in the left eye, demonstrating no clinically significant proptosis. Pupillary examination revealed no relative afferent pupillary defect (RAPD) in either eye. Dilated fundus examination showed clear vitreous and a cup-to-disc ratio of 0.3 in both eyes. There was no evidence of commotio retinae, retinal hemorrhage, or Berlin edema.

Neurosurgical management and clinical course

After neurosurgical review of the imaging findings, the patient underwent emergency craniotomy on the day of admission for evacuation of the epidural hematoma. Due to prolonged mechanical ventilation, a tracheostomy was performed approximately two weeks later. Over the following days, his neurological status gradually improved, reaching a GCS score of E4VTM6. During recovery in the intensive care unit, physicians noted a new onset of ocular misalignment of the right eye. Consequently, the ophthalmology and plastic surgery teams were re-consulted to evaluate the ocular deviation and reassess the previously identified facial fractures.

Repeat ophthalmic evaluation

At the time of re-evaluation, the patient was cooperative and able to undergo a complete ocular examination. Best-corrected visual acuity was 20/30 in both eyes using a near chart. The patient denied ocular pain or diplopia at this stage. Slit-lamp examination revealed no conjunctival injection, corneal injury, or anterior chamber abnormalities, and RAPD remained absent bilaterally. Confrontation visual field testing and color vision assessment were normal.

**Figure 2:**
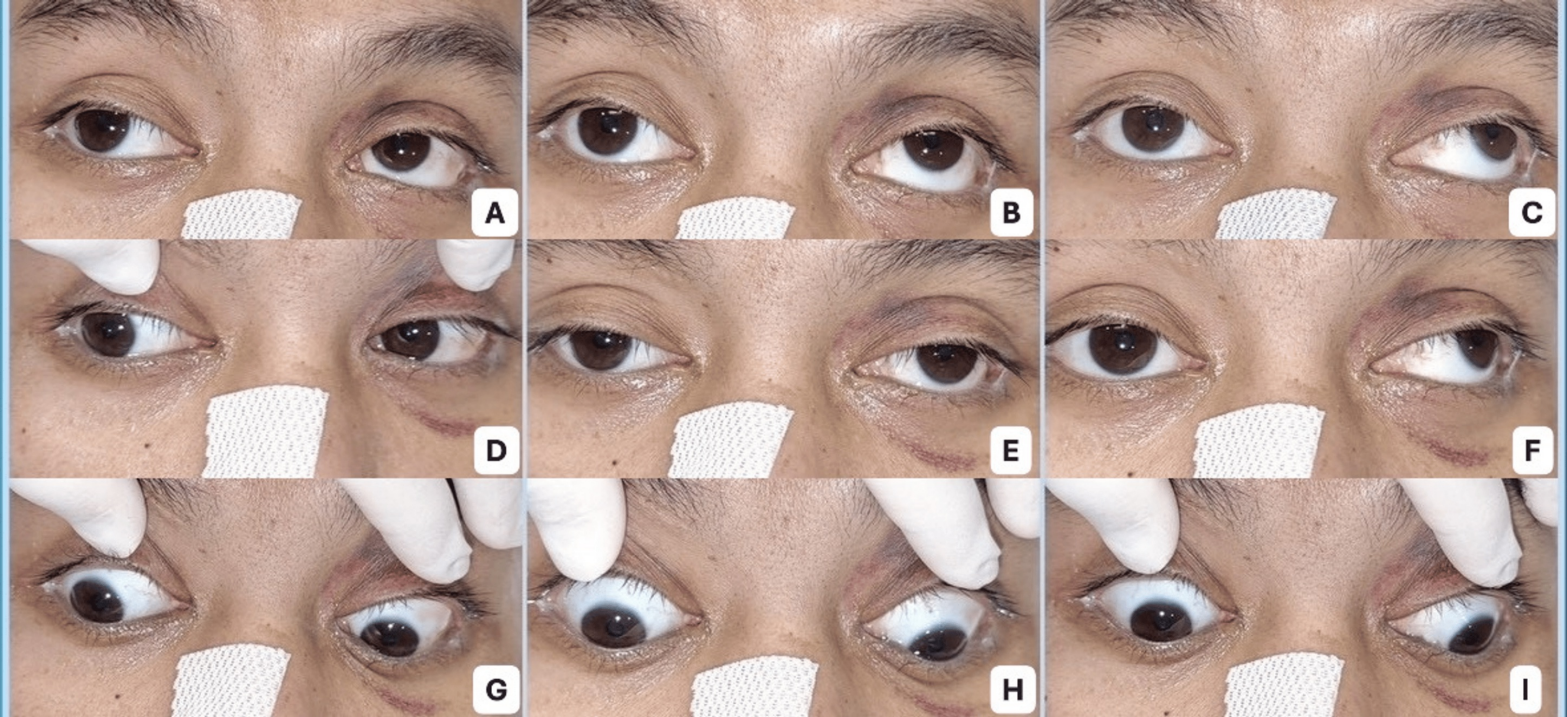
Ocular motility examination at the time of ophthalmology re-consultation. (A) Upgaze right, (B) upgaze primary, (C) upgaze left, (D) right gaze, (E) primary position, (F) left gaze, (G) downgaze right, (H) downgaze primary, and (I) downgaze left. The right eye demonstrates marked limitation of adduction that fails to cross the midline, particularly on left gaze (F), while the left eye exhibits abducting nystagmus on left gaze, producing an internuclear ophthalmoplegia (INO)-like pattern consistent with pseudo-internuclear ophthalmoplegia. Mild limitation of right eye upgaze is also observed.

Extraocular movement examination revealed a marked limitation of adduction in the right eye that failed to cross the midline. When the patient attempted leftward gaze, the left eye demonstrated abducting nystagmus, producing an ocular motility pattern highly suggestive of internuclear ophthalmoplegia. Additionally, mild limitation of right eye upgaze was observed. Although formal quantitative measurements, such as Hess charting, were not performed, the degree of adduction deficit was clinically significant, as the right eye failed to cross the midline. Importantly, convergence was preserved, and no other neurological deficits were identified, supporting a peripheral mechanism rather than a true medial longitudinal fasciculus lesion. The ocular motility findings are illustrated in Figure 2, particularly the marked limitation of right eye adduction and abducting nystagmus of the left eye on left gaze (Figure 2F), and a video recording of the eye movements is provided in Video 1.

**Video 1:** Ocular motility demonstrating pseudo-internuclear ophthalmoplegia pattern. This video demonstrates ocular motility findings at the time of ophthalmologic re-evaluation. The right eye shows marked limitation of adduction that fails to cross the midline, while the left eye exhibits abducting nystagmus during leftward gaze. This pattern mimics internuclear ophthalmoplegia and is consistent with pseudo-internuclear ophthalmoplegia in this clinical context.

Because of these findings, the neuro-ophthalmology service was consulted. Based on the clinical presentation and history of trauma, the differential diagnosis was narrowed to: true internuclear ophthalmoplegia due to medial longitudinal fasciculus (MLF) injury; pseudo-internuclear ophthalmoplegia secondary to traumatic medial rectus dysfunction; mechanical restriction from medial rectus entrapment associated with the medial orbital wall fracture; and neuromuscular junction disorders such as ocular myasthenia gravis. The preservation of convergence, absence of additional brainstem signs, and subsequent rapid clinical improvement were key distinguishing features favoring pseudo-internuclear ophthalmoplegia over true internuclear ophthalmoplegia. Further neuroimaging was planned to clarify the diagnosis.

Facial fracture repair and postoperative findings

**Figure 3:**
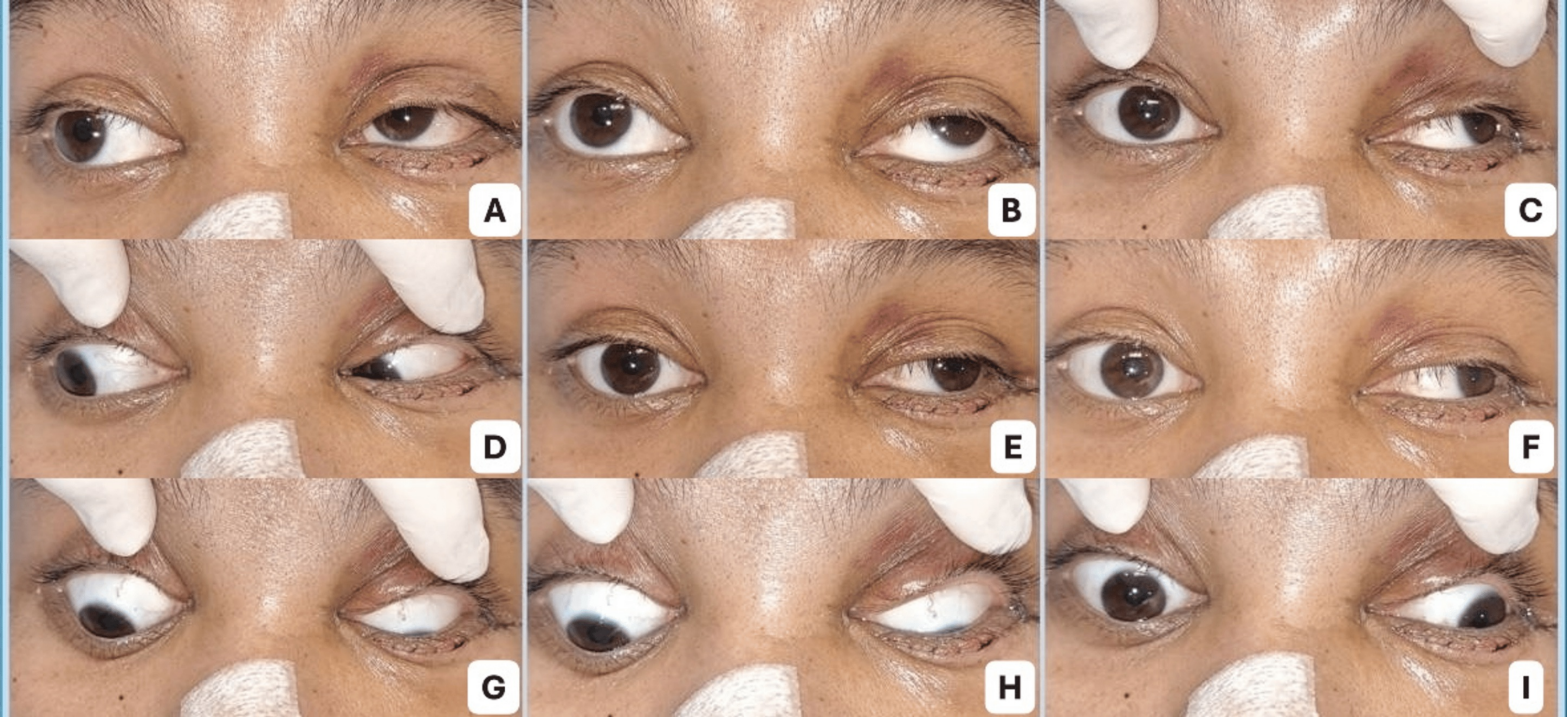
Ocular motility following facial fracture repair. (A) Upgaze right, (B) upgaze primary, (C) upgaze left, (D) right gaze, (E) primary position, (F) left gaze, (G) downgaze right, (H) downgaze primary, and (I) downgaze left. Persistent limitation of adduction of the right eye is observed, particularly on left gaze (F), where the eye fails to cross the midline. This is associated with diplopia and is consistent with an internuclear ophthalmoplegia (INO)-like pattern following orbital trauma.

The following day, the plastic surgery team performed open reduction and internal fixation of the left ZMC fracture using plates and screws, along with orbital floor reconstruction with intermaxillary fixation and closed reduction of the nasal fracture. Postoperative ophthalmic examination revealed BCVA reduced to 20/50 in both eyes. Extraocular movements remained abnormal (Figure 3), with persistent failure of right eye adduction beyond the midline, most evident on left gaze (Figure 3F).

At this stage, the patient reported new-onset diplopia on left gaze and mild ocular pain, symptoms that had not been present during the earlier examination. Because of the persistent ocular motility deficit, additional imaging was planned. CT of the orbit was initially considered but was avoided due to potential artifact from the recently placed metallic hardware. Instead, magnetic resonance imaging (MRI) of the brain and orbits with gadolinium contrast was scheduled to evaluate for subtle brainstem injury or extraocular muscle pathology.

Spontaneous clinical improvement

**Figure 4:**
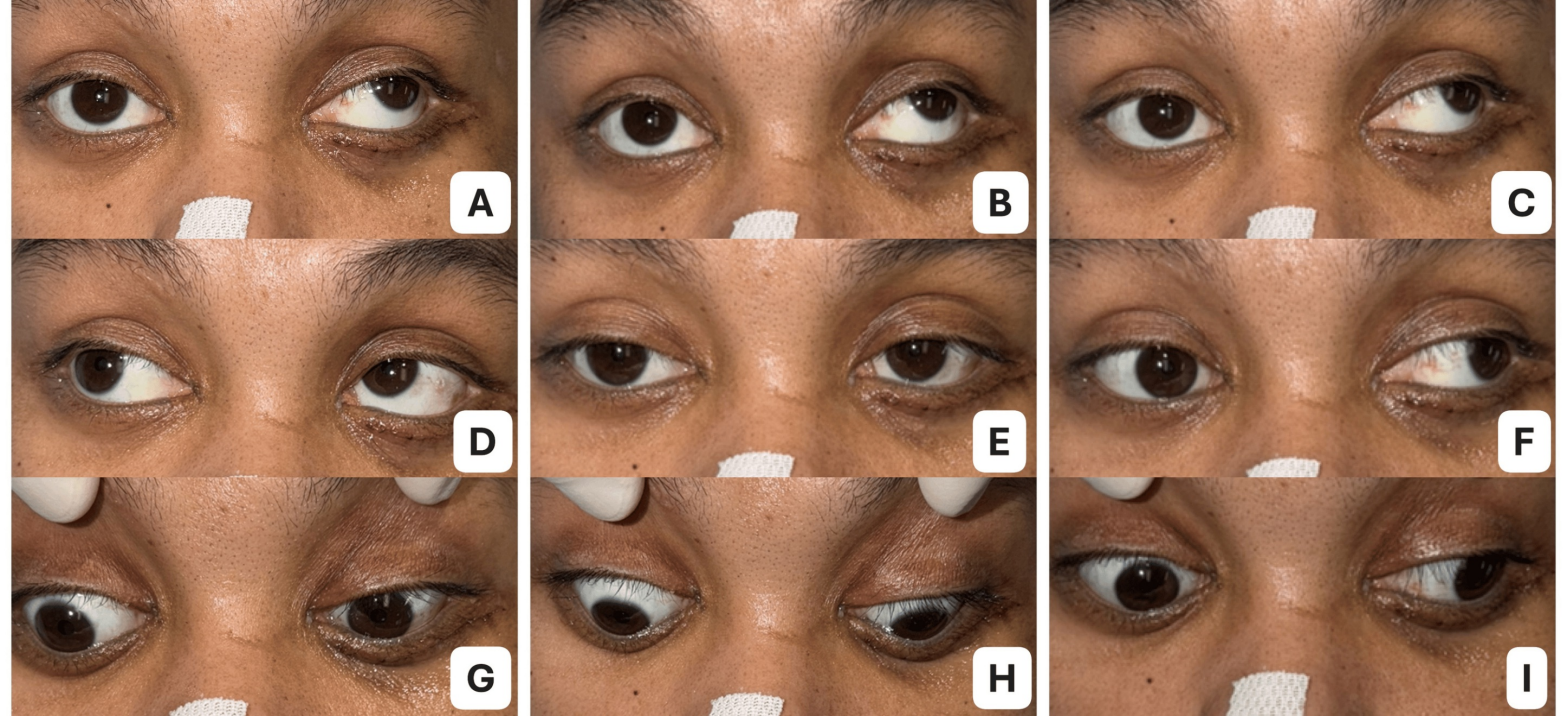
Follow-up ocular motility demonstrating spontaneous recovery. (A) Upgaze right, (B) upgaze primary, (C) upgaze left, (D) right gaze, (E) primary position, (F) left gaze, (G) downgaze right, (H) downgaze primary, and (I) downgaze left. Improvement of right eye adduction is observed, particularly on left gaze (F), where the eye is now able to cross the midline to approximately 80% of the normal range. This clinical recovery supports transient medial rectus dysfunction rather than a structural brainstem lesion.

However, several days before the scheduled MRI, approximately one week after the facial fracture surgery, substantial spontaneous improvement was observed during routine ward rounds. Best-corrected visual acuity improved to 20/30 in the right eye and 20/40 in the left eye. The patient reported resolution of ocular pain, and diplopia was now present only in extreme left gaze. Extraocular motility of the right eye showed marked recovery. The eye could now cross the midline to approximately 80% of the normal adduction range during left gaze, and right eye upgaze improved to approximately 50% of the normal range (Figure 4).

Given the rapid spontaneous improvement and absence of radiologic evidence of brainstem injury, the diagnosis was revised to pseudo-internuclear ophthalmoplegia secondary to traumatic medial rectus dysfunction, likely caused by muscle contusion associated with the subtle medial orbital wall fracture. Because of the significant clinical improvement, the previously planned MRI examination was deferred. The patient was discharged with topical antibiotic therapy and scheduled for follow-up evaluation in the ophthalmology strabismus clinic within three months.

Timeline

The timeline of clinical history is summarized in Table 1.

**Table 1:** Timeline of clinical history.

Time	Event
Day 0	Motorcycle accident with loss of consciousness
Day 0	CT of the brain and facial bones performed
Day 1	Initial ophthalmology consultation
Post-op week 1	Spontaneous improvement in ocular motility
Week 2	Diplopia improved; discharge from hospital

Follow-up and outcomes

Two weeks after the initial ophthalmic evaluation, ocular motility demonstrated substantial improvement. The right eye could adduct to approximately 80% of the normal range beyond the midline. The patient reported gradual resolution of diplopia, which was present only in extreme left gaze at the time of discharge.

## Discussion

Internuclear ophthalmoplegia (INO) is a characteristic ocular motor syndrome caused by disruption of the medial longitudinal fasciculus (MLF), a brainstem pathway that connects the abducens nucleus to the contralateral oculomotor nucleus and coordinates conjugate horizontal gaze [1,2]. Damage to this pathway typically produces a recognizable clinical pattern consisting of adduction deficit of the ipsilateral eye, accompanied by abducting nystagmus of the contralateral eye during horizontal gaze, often with preserved convergence [2]. Because the MLF lies within the dorsal brainstem, INO has traditionally been regarded as a highly localizing sign of central nervous system pathology.

The underlying causes of INO vary according to patient age and clinical context. The most common etiologies include ischemic stroke in older patients and demyelinating disease, particularly multiple sclerosis, in younger individuals [3]. Less frequent causes include trauma, tumors, infections, inflammatory conditions, and metabolic disorders [3]. In a large clinical series reported by Keane, traumatic causes represented a minority of cases among patients with INO but remained an important diagnostic consideration, particularly in individuals presenting after head injury [3].

In contrast, pseudo-internuclear ophthalmoplegia refers to an INO-like ocular motility pattern occurring in the absence of a structural lesion of the MLF. Several peripheral mechanisms have been described that may reproduce the clinical appearance of INO. The most frequently reported cause is ocular myasthenia gravis, in which fatigable weakness of the medial rectus muscle produces impaired adduction that can mimic true INO [5,6]. Similar pseudo-INO patterns have also been described in Guillain-Barré syndrome and other neuromuscular disorders affecting ocular motor function [7]. In such conditions, medial rectus dysfunction results in increased neural drive to the horizontal gaze system during attempted lateral gaze, which may generate contralateral abducting nystagmus that resembles the ocular motor pattern of true INO.

Pseudo-INO may also arise from mechanical or iatrogenic impairment of the medial rectus muscle. von Noorden and colleagues first described pseudo-internuclear ophthalmoplegia following surgical weakening of the medial rectus muscle during strabismus surgery [4]. In these cases, patients developed adduction deficits with contralateral abducting nystagmus despite the absence of brainstem pathology, demonstrating that peripheral medial rectus weakness alone can reproduce the characteristic ocular motor features of INO [4]. These observations suggest that disruption of medial rectus function may alter the balance of neural input within the horizontal gaze network, producing compensatory eye movement patterns that resemble those seen in central lesions.

Trauma-related pseudo-INO has been reported only rarely in the literature. Most traumatic cases of INO described previously represent true INO due to structural injury of the MLF, typically confirmed by magnetic resonance imaging demonstrating focal brainstem lesions [11]. For example, traumatic INO following mild traumatic brain injury has been reported with neuroimaging evidence of brainstem involvement [11]. In contrast, reports describing pseudo-INO resulting from extraocular muscle injury or orbital trauma are extremely limited, making the present case of particular clinical interest.

Several findings support a peripheral rather than central mechanism in this case. Neuroimaging showed no evidence of brainstem or medial longitudinal fasciculus involvement, while orbital imaging demonstrated a subtle medial orbital wall fracture adjacent to the medial rectus muscle, providing a plausible anatomical basis for the adduction deficit. In addition, the rapid spontaneous improvement in ocular motility within two weeks is more consistent with transient muscle contusion, edema, or neuropraxia than with structural brainstem injury. Alternative diagnoses were considered and systematically excluded: true internuclear ophthalmoplegia was unlikely given the absence of additional neurological deficits and the favorable clinical course; mechanical entrapment was not supported by imaging findings; and neuromuscular junction disorders such as ocular myasthenia gravis were considered less likely due to the acute traumatic onset and non-fluctuating presentation.

Blunt orbital trauma may impair extraocular muscle function through multiple mechanisms, including direct muscle contusion, ischemia, partial muscle rupture, nerve injury, or mechanical entrapment within orbital fractures [8]. Although orbital floor fractures are more commonly associated with inferior rectus entrapment, medial orbital wall fractures may involve the medial rectus muscle and result in clinically significant ocular motility disturbances, even in the absence of overt radiologic entrapment [9,12]. Importantly, even subtle fractures may cause temporary muscle dysfunction due to local inflammation, edema, or transient impairment of muscle contractility.

Another notable feature of this case is the absence of radiologic evidence of medial rectus entrapment despite a significant adduction deficit. This observation suggests that transient medial rectus contusion or localized muscle injury, rather than mechanical incarceration, was responsible for the ocular motility disturbance. The gradual resolution of symptoms and improvement in ocular motility further support this interpretation.

Recent reviews of traumatic ocular motility disorders emphasize that extraocular muscle injury may occasionally produce complex ocular motor patterns that mimic central gaze abnormalities, highlighting the importance of careful clinical localization in trauma patients [8]. In particular, involvement of the medial rectus muscle in medial orbital wall fractures has increasingly been recognized as a clinically important mechanism of horizontal diplopia. Contemporary studies indicate that medial rectus dysfunction may occur even in the absence of obvious muscle entrapment on imaging, particularly when fractures are small or minimally displaced [12].

Reports describing pseudo-internuclear ophthalmoplegia caused by traumatic medial rectus dysfunction without entrapment remain extremely rare. Most previously reported pseudo-INO cases involve neuromuscular disorders such as myasthenia gravis or iatrogenic medial rectus weakness following strabismus surgery [4-6]. The present case, therefore, contributes additional evidence that orbital trauma with a subtle medial wall fracture may mimic internuclear ophthalmoplegia through transient medial rectus dysfunction.

Recognition of this mechanism has important clinical implications. Misinterpretation of such ocular motility findings as true INO may lead to mislocalization of the lesion to the brainstem and unnecessary neurological investigations. In patients presenting with INO-like ocular motor patterns after facial trauma, careful assessment of orbital imaging, extraocular muscle function, and clinical course is essential to differentiate central from peripheral causes.

This case has several limitations. Firstly, magnetic resonance imaging (MRI) was not conducted, which hampers our ability to definitively rule out a subtle lesion in the medial longitudinal fasciculus. The diagnosis of medial rectus dysfunction relied on clinical and radiological correlation without direct confirmation. Furthermore, since this is a single case study, the findings may not be applicable to a broader population and should be viewed as generating hypotheses rather than definitive conclusions. Lastly, the long-term follow-up was limited to the acute recovery period, which restricts our ability to assess sustained functional outcomes.

## Conclusions

Internuclear ophthalmoplegia is typically associated with lesions of the medial longitudinal fasciculus within the brainstem. However, peripheral orbital pathology may occasionally produce an INO-like ocular motility pattern. We report a rare case of traumatic pseudo-internuclear ophthalmoplegia most likely resulting from transient medial rectus dysfunction associated with a subtle medial orbital wall fracture. Although magnetic resonance imaging was not performed to definitively exclude a subtle medial longitudinal fasciculus lesion, the clinical findings and rapid spontaneous improvement strongly support a peripheral mechanism. Recognition of this atypical presentation is important to avoid mislocalization of the lesion and unnecessary neurological investigations in trauma patients presenting with INO-like ocular motility findings.

## References

[1] Feroze KB, Wang J (2025). Internuclear ophthalmoplegia. StatPearls [Internet].

[2] Lee AG (2026). EyeWiki: Internuclear ophthalmoplegia. https://eyewiki.org/Internuclear_Ophthalmoplegia.

[3] Keane JR (2005). Internuclear ophthalmoplegia: unusual causes in 114 of 410 patients. Arch Neurol.

[4] Von Noorden GK, Tredici TD, Ruttum M (1984). Pseudo-internuclear ophthalmoplegia after surgical paresis of the medial rectus muscle. Am J Ophthalmol.

[5] McClard CK, Lyons LJ, Yalamanchili S (2018). Bilateral pseudo-internuclear ophthalmoplegia in a patient with myasthenia gravis. Am J Ophthalmol Case Rep.

[6] Munasinghe KV, Herath WA, Silva FH (2023). A case report on pseudo-internuclear ophthalmoplegia: a rare manifestation of myasthenia gravis. Cureus.

[7] Diamond S, Schear HE, Leeds MF (1975). Pseudo-internuclear oculomotor ophthalmoplegia secondary to Guillain-Barré polyneuronitis simulating myasthenia gravis in a air transport pilot. Aviat Space Environ Med.

[8] Lin CH, Lin PC, Hsieh CH (2025). Extraocular muscle trauma: clinical approach to diagnosis and surgical management of rectus muscle disruptions. World J Plast Surg.

[9] Thiagarajah C, Kersten RC (2009). Medial wall fracture: an update. Craniomaxillofac Trauma Reconstr.

[10] Teasdale G, Jennett B (1974). Assessment of coma and impaired consciousness: a practical scale. Lancet.

[11] Hai S, Elkbuli A, Kinslow K, McKenney M, Boneva D (2019). When "looks" can be deceiving: internuclear ophthalmoplegia after mild traumatic brain injury: case report and literature review. Int J Surg Case Rep.

[12] Holzmer S, O'Rorke E, Martin M, Gupta S (2025). Navigating the rare medial rectus entrapment in orbital fractures: a 5-year series and systematic review of the literature. Ann Plast Surg.

